# Backbone ^1^H, ^13^C, and ^15^N resonance assignments of the Fc fragment of human immunoglobulin G glycoprotein

**DOI:** 10.1007/s12104-014-9586-7

**Published:** 2014-10-08

**Authors:** Hirokazu Yagi, Ying Zhang, Maho Yagi-Utsumi, Takumi Yamaguchi, Shigeru Iida, Yoshiki Yamaguchi, Koichi Kato

**Affiliations:** 1 Graduate School and Faculty of Pharmaceutical Sciences, Nagoya City University, Nagoya, 467-8603 Japan; 2 Okazaki Institute for Integrative Bioscience and Institute for Molecular Science, National Institutes of Natural Sciences, Okazaki, Aichi 444-8787 Japan; 3 Kyowa Hakko Kirin, Co., Ltd., Chiyoda-ku, Tokyo 100-0004 Japan; 4 Structural Glycobiology Team, Systems Glycobiology Research Group, RIKEN-Max Planck Joint Research Center, RIKEN Global Research Cluster, 2-1 Hirosawa, Wako, Saitama 351-0198 Japan

**Keywords:** Immunoglobulin G, Fc, Glycoprotein, Mammalian expression system, NMR spectroscopy, Resonance assignment

## Abstract

The Fc portion of immunoglobulin G (IgG) recruits complements and its cognate receptors, thereby promoting defensive mechanisms in the humoral immune system. These effector functions critically depend on *N*-glycosylation at the Fc region, which is therefore regarded as a crucial factor in the design and production of therapeutic antibodies. NMR spectroscopy plays a unique role in the characterization of conformational dynamics and intermolecular interactions of IgG-Fc in solutions. Here, we report NMR assignments of the glycosylated Fc fragment (*M*r 53 kDa), cleaved from a chimeric antibody with human IgG1 constant regions, which was produced in Chinese hamster ovary cells with uniform ^13^C- and ^15^N-labeling.

**Electronic supplementary material:**

The online version of this article (doi:10.1007/s12104-014-9586-7) contains supplementary material, which is available to authorized users.

## Biological context

Immunoglobulin G (IgG) is a multifunctional glycoprotein composed of an Fc region and two Fab regions, which are connected through the hinge region (Yamaguchi et al. 2007). The Fab regions recognize and capture specific antigens, while the Fc region recruits complements and its cognate receptors, Fcγ receptors (FcγRs), and offers acceptor sites for bacterial proteins including protein A and protein G. The Fc region has a homodimeric structure comprising the C-terminal halves of the heavy chains, each composed of the C_H_2 and C_H_3 domains. The C_H_2 domain possesses a conserved *N*-glycosylation site, Asn297, at which a biantennary complex-type oligosaccharide is expressed with microheterogenieties characterized by the presence and absence of the non-reducing terminal galactose, fucose, sialic acid, and bisecting *N*-acetylglucosamine residues.

The effector function of IgG critically depends on *N*-glycosylation in the Fc region. The outer carbohydrate moieties govern the structural integrity of the FcγR-binding site of IgG, while the core fucosylation impairs antibody-dependent cellular cytotoxicity because of its negative steric effect against IgG interaction with FcγRIII (Ferrara et al. 2011; Krapp et al. 2003; Mizushima et al. 2011; Yamaguchi et al. 2006). Hence, the Fc glycoforms are now considered as a crucial factor in the design and production of therapeutic antibodies in biopharmaceutical fields (Berkowitz et al. 2012; Jiang et al. 2011).

NMR spectroscopy offers unique tools for characterizing the conformational dynamics and intermolecular interactions of IgG-Fc in solution (Kato et al. 1991a, 1993a, 1995; Kim et al. 1994a; Latypov et al. 2012). We developed protocols for uniform and amino acid-selective stable isotope labeling of an IgG glycoprotein and its functional fragments, using mammalian expression systems (Kato et al. 2010; Yamaguchi and Kato 2010). Based on partially (approximately 66 %) achieved spectral assignments (Yamaguchi et al. 2006), we previously reported NMR analytical results to characterize the *N*-glycosylation-dependent conformational changes of human IgG1-Fc and its interaction with a specific RNA aptamer (Matsumiya et al. 2007; Miyakawa et al. 2008; Yamaguchi et al. 2006).

In an extension of these studies, we herein report NMR assignments of the glycosylated version of Fc fragment (*M*r 53 kDa) cleaved from a chimeric antibody with human IgG1 constant regions that was expressed by Chinese hamster ovary (CHO) cells with uniform ^13^C- and ^15^N-labeling.

## Methods and experiments

The CHO/DG44 cell line (Urlaub1980) was kindly provided by Dr. Lawrence Chasin (Columbia University, NY). An anti-CCR4 chimeric antibody (designated KM3060), with human IgG1/κ constant regions, was produced in a CHO cell line as described previously (Yamaguchi and Kato 2010; Yamaguchi et al. 2006). The CHO cells were cultivated using the Nissui NYSF 404 medium supplemented with 2 % dialyzed fetal bovine serum. At the final stage of cell culture, the medium was replaced with an isotopically labeled one with 2 % dialyzed fetal bovine serum. Uniformly ^15^N/^13^C-labeled IgG1 was prepared using a modified Nissui NYSF 404 medium (supplemented with 2 % dialyzed fetal bovine serum) in which glucose, sodium pyruvate, succinic acid, and amino acids were replaced by 1 g/L [^15^N/^13^C]algal amino acid mixture, 2 g/L D-[^13^C_6_]glucose, 110 mg/L [^13^C_3_]pyruvic acid sodium salt, 59 mg/L [^13^C_4_]succinic acid, 149 mg/L L-[^13^C_6_,^15^N_4_]Arg·HCl, 42.5 mg/L L-[^13^C_4_,^15^N_2_]Asn·H_2_O, 24 mg/L L-[^13^C_3_,^15^N]Cys, 450 mg/L L-[^13^C_5_,^15^N_2_]Gln, 17 mg/L L-[^13^C_6_,^15^N_3_]His·HCl·H_2_O, 27 mg/L L-[^13^C_9_,^15^N]Tyr, and 7 mg/L L-[^13^C_11_,^15^N_2_]Trp. Amino acid-selective labeling of IgG1 was performed using the modified Nissui NYSF 404 medium (supplemented with 2 % dialyzed fetal bovine serum) in which selected amino acid components were substituted with isotopically labeled analogs, as described previously (Kato et al. 1991a, b, 1993b; Kim et al. 1994b). After cell growth, the supernatant was purified using an Affi-gel protein A column (GE Healthcare Bio-Sciences), as described previously (Yamaguchi et al. 1995). The Fc fragment of IgG1 was prepared by papain digestion, performed at 37 °C for 12 h in 75 mM sodium phosphate buffer (pH 7.0) containing 75 mM NaCl, and 2 mM EDTA. The protein concentration was 10 mg/ml, and the ratio of papain/IgG1 was 1:50 (w:w). The digestion products were loaded onto an Affi-gel protein A column. To prepare IgG1-Fc exhibiting a homogeneous *N*-glycan, GlcNAcβ1-2Manα1-3(GlcNAcβ1-2Manα1-6)Manβ1-4GlcNAcβ1-4(Fucα1-6)GlcNAc, the Fc fragment was treated with a recombinant *Streptococcus pneumoniae* β1,4-galactosidase (CALBIOCHEM), according to the literature (Yamaguchi et al. 2006).

For NMR measurements, the Fc fragment was dissolved in 0.5 ml of 5 mM sodium phosphate buffer (pH 6.0) containing 50 mM NaCl and 10 % (v/v) D_2_O. NMR spectra were acquired at 42 or 52 °C using DMX500 (Bruker BioSpin), AVANCE800 (Bruker BioSpin), and ECA-920 (JEOL) spectrometers. Chemical shifts of ^1^H were referenced to DSS (0 ppm), and ^13^C and ^15^N chemical shifts were referenced indirectly using the gyromagnetic ratios of ^13^C, ^15^N, and ^1^H (*γ* ^13^C/*γ* ^1^H = 0.25144952; *γ* ^15^N/*γ* ^1^H = 0.10132905).

Backbone resonance assignments were made on the basis of 2D ^1^H–^15^N HSQC spectral data of uniformly or selectively ^13^C/^15^N-labeled IgG1-Fc, and 3D spectral data obtained with the following experiments: HNCA, HNCO, HN(CA)CO, CBCA(CO)NH, and HNCACB. All NMR data were processed using NMRPipe software (Delaglio et al. 1995), and analyzed with SPARKY (Goddard and Kneller 1993) and CcpNmr (Vranken et al. 2005) software.

## Assignments and data deposition

**Fig. 1:**
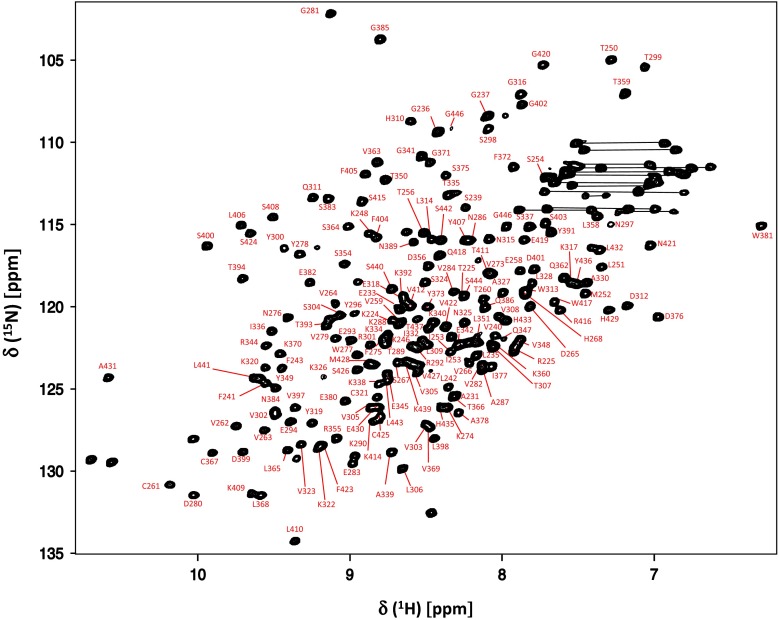
^1^H–^15^N HSQC spectrum of uniformly ^13^C, ^15^N-labeled IgG-Fc recorded at 52 °C. Backbone assignments are annotated by the resonance peaks with one-letter amino acid codes and the sequence numbers. Side-chain resonances corresponding to NH_2_ amides are connected by *horizontal lines*

Figure 1 shows the ^1^H–^15^N HSQC spectrum of human IgG1-Fc. Although the use of a mammalian expression system is mandatory for preparing antibodies with physiological glycosylation, uniform deuteration of the glycoprotein is not facile in such a system (Liu et al. 2007). Hence, we established spectral assignments based on the triple resonance spectral dataset recorded at a higher temperature, i.e. 52 °C, complemented with HSQC spectral data obtained by amino acid-selective ^13^C/^15^N-labeling. Chemical shift assignments were made for protein backbone resonances: Cα (99 %), Cβ (84 %), CO (80 %), HN (99 %), and N (99 %) (except for N of prolines). The spectral assignments at lower temperatures could be extrapolated by observing progressive spectral changes, depending on temperature, as exemplified by the spectrum at 42 °C (Supplemental Fig.1). The present spectral assignments indicate that a cluster of amino acid residues in the vicinity of the *N*-glycans, i.e. Gln295-Thr299 exhibit significant chemical shift differences in comparison with the previously reported assignments of human Fc produced in Escherichia coli (Liu et al. 2007).

The assignments for the ^1^H, ^13^C, and ^15^N backbone resonances of human IgG1-Fc have been deposited in the BioMagResBank database (http://www.bmrb.wisc.edu) under the accession number 25224.

## Electronic supplementary material

Below is the link to the electronic supplementary material.

Supplementary material 1 (PPT 615 kb)
