# Supplementary material for: Backbone 1H, 13C, and 15N resonance assignments of the Fc fragment of human immunoglobulin G glycoprotein
Source: Biomol NMR Assign. 2014 Oct 8;9(2):257–60. doi: 10.1007/s12104-014-9586-7 (PMC4568019; doi:10.1007/s12104-014-9586-7)
Supplement: Supplementary file 1 — Supplementary material 1 (PPT 615 kb) [file 12104_2014_9586_MOESM1_ESM.ppt]

## Slide 1
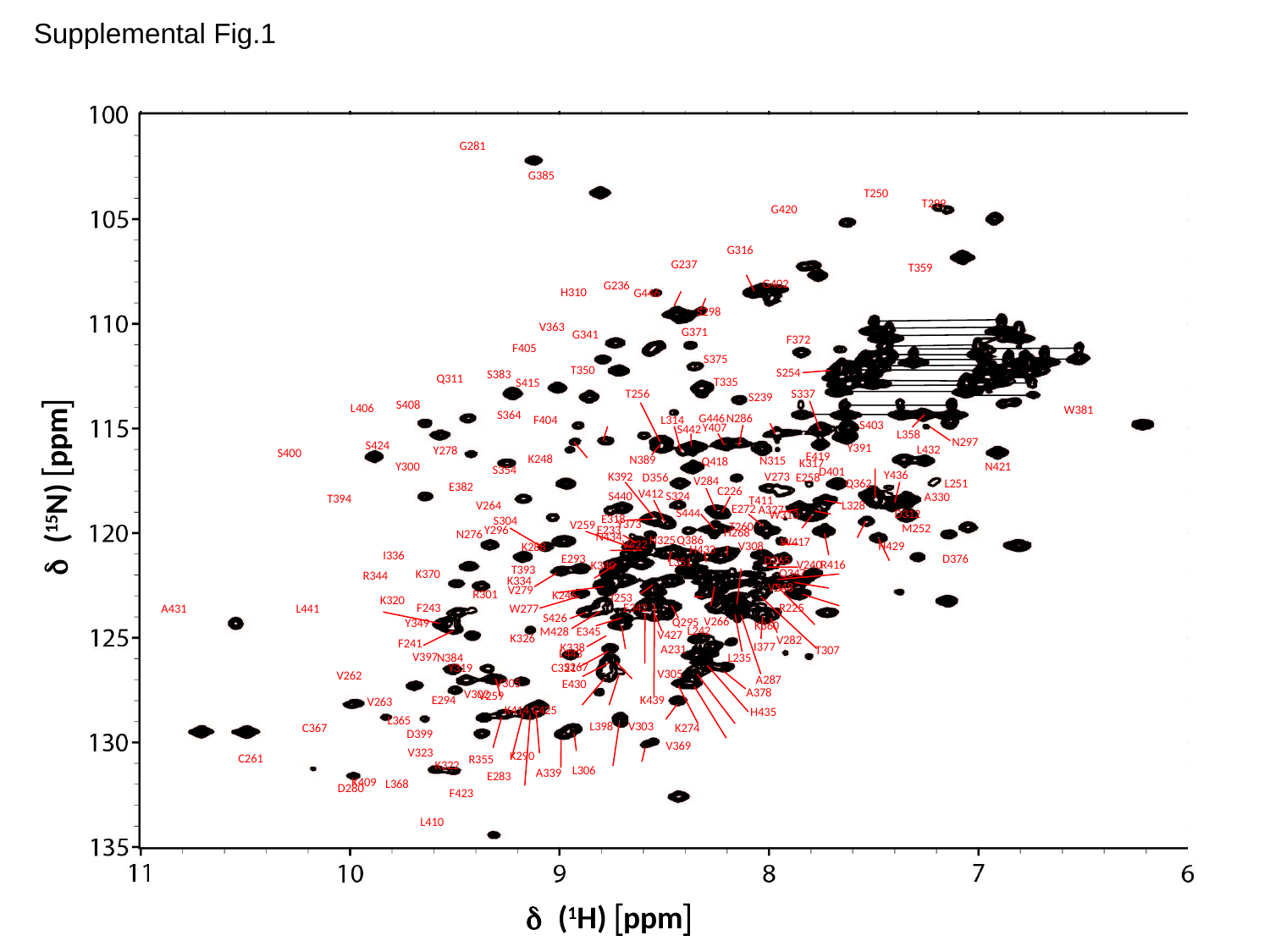

Supplemental Fig.1
G281
G385
T250
T299
G420
G316
G237
T359
G402
G236
H310
G446
S298
V363
G371
G341
F372
F405
S375
T350
S254
S383
Q311
T335
S415
T256
S337
S239
S408
L406
W381
S364
G446
N286
L314
F404
S403
Y407
S442
 (15N) ppm
L358
N297
S424
Y391
L432
Y278
S400
E419
K248
N389
N315
Q418
K317
N421
Y300
S354
D401
Y436
K392
V273
D356
E258
V284
Q362
L251
E382
C226
V412
S440
S324
A330
T394
T411
L328
V264
E272
A327
S444
D312
W313
E318
S304
Y373
V259
T260
M252
Y296
E233
H268
N276
N434
N325
Q386
W417
V422
V308
H429
K288
H433
I336
D376
E293
D265
L351
V240
R416
K340
T393
Q347
K370
R344
K334
V348
V279
R301
K246
I253
K320
E342
R225
F243
A431
L441
W277
S426
V266
Q295
Y349
K360
L242
E345
M428
V427
K326
V282
F241
I377
K338
A231
T307
L443
V397
N384
L235
S267
C321
Y319
V305
V262
A287
V305
E430
A378
V302
V259
E294
K439
V263
K414
C425
H435
L365
L398
V303
C367
K274
D399
V369
V323
K290
C261
R355
K322
L306
A339
E283
K409
L368
D280
F423
L410
 (1H) ppm
